# Patient Use of Email for Health Care Communication Purposes Across 14 European Countries: An Analysis of Users According to Demographic and Health-Related Factors

**DOI:** 10.2196/jmir.3700

**Published:** 2015-03-06

**Authors:** Nikki Newhouse, Francisco Lupiáñez-Villanueva, Cristiano Codagnone, Helen Atherton

**Affiliations:** ^1^Nuffield Department of Primary Care Health SciencesUniversity of OxfordOxfordUnited Kingdom; ^2^UCL Interaction CentreUniversity College LondonLondonUnited Kingdom; ^3^Department of Information and Communication ScienceUniversitat Oberta de CatalunyaBarcelonaSpain; ^4^Applied Social Science and Behavioural Economics Research GroupUniversitat Oberta de CatalunyaBarcelonaSpain; ^5^Dipartimento di Scienze Sociali e PoliticheUniversitá degli Studi di MilanoMilanItaly

**Keywords:** eHealth, patient-doctor communication, electronic mail, Internet, Europe, chronic illness, patient activation, health services, survey

## Abstract

**Background:**

The use of the Internet for health purposes is growing steadily, yet the use of asynchronous communication tools for health care purposes remains undeveloped. The introduction of email as a method of communication in health care has the potential to impact on both patients and health care professionals.

**Objective:**

This study aims to describe the characteristics of people who have sent or received an email to or from their doctor, nurse, or health care organization, by country and in relation to demographics, health care resource use, and health status factors.

**Methods:**

We conducted a secondary analysis of data (N=14,000) collected from the online Citizens and Information Communication Technology for Health survey, a project undertaken in 2011 by the Institute for Prospective Technology Studies of the European Commission’s Joint Research Centre. The survey was developed to understand and characterize European citizens’ use of information communication technologies for health. Descriptive and statistical analyses of association were used to interpret the data.

**Results:**

Denmark reported the highest level of emails sent/received (507/1000, 50.70%). The lowest level reported was by participants in France (187/1000, 18.70%). Men used email communication for health care more than women, as did respondents in the 16-24 age group and those educated to tertiary level or still within the education system. As self-reported health state worsens, the proportion of people reporting having sent or received an email within the context of health care increases. Email use, poor health, multimorbidity, and number of visits to a physician are positively correlated.

**Conclusions:**

The use of email communication within the context of European health care is extremely varied. The relationship between high email use, poor health, doctor visits, and multimorbidity is especially pertinent: provision of asynchronous communication for such groups is favored by policymakers. Low reported email use by country may not necessarily reflect low interest in using email for health care: local health policies and technical infrastructures may be significant factors in the delay in implementation of alternative forms of routine health communication.

## Introduction

### Background

The use of the Internet for health purposes is growing steadily as people increasingly go online to access factual and experiential information and to share their own health and illness experiences [1-3]. While health-related Internet use has increased steadily, the use of asynchronous communication tools for health care purposes among European populations remains relatively undeveloped and unexplored. Asynchronous communication refers to interaction that is non-concurrent, such as email, as opposed to the synchronous, real-time communication offered by phone consultation, for example. Email is a commonly used method of communication globally. It has become a major element of day-to-day life for many people, both at work and in their personal lives. However, the uptake in the health care sector has been much lower than for other sectors, and email is not routinely used as a way for patients to contact their health care organization or professional [4,5] despite a reportedly high interest in associated eHealth services among European citizens [6]. Various national policies encourage email use by patients in the health care setting [7-11], for example in Denmark, where patients are able to engage in electronic communication with their family doctor via the official Danish health website [12]. In the United States, health maintenance organizations such as Kaiser Permanente have embraced email for communicating with their patients, offering patient portals where patients can log in and use Web messaging to send an email to their health care professional, make appointments, and receive test results [13,14]. At present, relatively little is known about how patients use email to interact with health care, but this information is likely to be valuable in determining the success of proposed policies and impact of email use. Key variables that may influence the uptake of email communication between patient and health professional include patient health status, the “digital divide”, and the potential impact on the use of health care services.

### Health Status

A recent study of the effect of asynchronous communication between health care providers and chronically ill patients found positive effects on health behavior, health outcomes, and patient satisfaction [15]. Indeed, it is well established that health status is directly connected to certain health-related online behaviors. Although individuals with chronic or multimorbid health conditions are less likely to have access to the Internet and to be less able to use it, evidence suggests that they are more likely to be engaged with the self-management of their own health [16,17]. Individuals with chronic and multimorbid health conditions are more likely to gather and share information about their health and to follow it up by seeking advice from a health professional, friend, family member, or peer [2]. The number of people living with chronic illnesses is increasing exponentially, and they are a primary target group for policy makers. They are also the group who have perhaps the most to gain from asynchronous communication opportunities due to their need for frequent contact with health care professionals.

The perception by health care professionals is that the open channel of communication offered by email may become congested by the “worried well”, those who value convenient access but have a substantially lesser need for this access than those suffering from multimorbidities [18]. This has the potential to impact on both utilization of health resources and overall clinician workload [19] and may be a key factor in clinicians’ reluctance to engage with asynchronous communication.

### The Digital Divide

The digital divide in the context of using email for health care communication refers to the opportunity gap that emerges when a service is available to those with access to the Internet and online services, in this case email, and unavailable to those who do not have this access. However, the resulting divide is nuanced beyond *having* and *not having* access: even among regular Internet users there is a recognized division in relation to *how* people use the Internet and for what purposes, for instance in how frequently they can or want to access the Internet and their level of computer literacy [20]. Although evidence is mixed, certain groups are deemed to be at an immediate disadvantage: older people, those with lower levels of education, and those not in regular employment [21,22].

As stated above, those with multimorbidities may benefit from having an additional communication route. But as multimorbidity and chronic illness may be more likely to affect older age groups, it is unclear whether this group could adequately benefit since older people are perceived to be less likely to want to engage with the Internet for health care purposes [23]. At present, little is known about the impact on this divide of introducing email as a method of communication in health care, despite this information having a potential impact on how health care is distributed.

### Health Care Utilization

There is the possibility that introducing an additional channel for communication in health care may lead to an increased number of contacts with health care services. Where email provides a communication directly with the health care professional, there is mixed evidence on how introducing it impacts on numbers of contacts by patients with their health care providers. It is widely stated by policy makers that introduction of these alternative methods of communication will reduce other types of contacts, and there is some evidence to support this [24]. However, there is also evidence showing that introduction of email for communicating with a health care professional increases the overall number of contacts by patients across other methods of communication [25]. At present, little is known about the behaviors of patients using email for their health care, in relation to visits made. This information is important in relation to planning of workload and funding, as well as determining whether the service brings what it intends to.

### Aim of the Study

The introduction of email as a method of communication in health care, be it between a patient and health care professional, or between patient and health care organization, has the potential to impact greatly on both patients and health care professionals by influencing the use of health systems, the relationship between patient and doctor, and the ways in which people manage their own health. An evidence-based understanding of current practice and trends in this area is crucial as we transition towards a future in which patients’ remote access to health care and encouragement of large-scale self-management will be policy priorities for many countries. This study describes the characteristics of an Internet-using population who have sent or received an email to or from their doctor, nurse, or health care organization, by country and in relation to demographic and health care resource use and health status factors.

## Methods

### Survey Instruments and Ethics

We conducted a secondary analysis of data collected from the *Citizens and Information Communication Technology for Health* survey, a project undertaken in 2011 by the Institute for Prospective Technology Studies of the European Commission’s Joint Research Centre. This online survey was developed from a theoretical framework of the social determinants of information and communication technology for health, translated into native languages in 14 European Union member countries. The survey was developed to understand and characterize European citizens’ use of information and communication technology for health. Technical, methodological, and legal considerations were carefully addressed in the context of designing and implementing the survey. These considerations ensured anonymity and confidentiality of individual responses [26-28]. The survey was conducted in accordance with European Society for Opinion and Marketing Research ethical guidelines [29]. At the time the survey was carried out, 2 of the authors (FLV, CC) were employed by the European Commission.

The questionnaire was structured in 5 blocks—Block A: Health status and health care and social care services use; Block B: Health attitude and Health information sources; Block C: Internet and Information and Communication Technologies uses; Block D: Health-related use of Information and Communication Technologies and the Internet; and Block E: Sociodemographic profile of participants.

### Sample and Data Collection

The target population was citizens aged 16-74 years old who had used the Internet in the previous 3 months. The survey was conducted online in Austria, Belgium, Germany, Denmark, Estonia, Finland, France, Italy, Netherlands, Sweden, Slovenia, Slovakia, Spain, and the United Kingdom with a proportional allocation of 1000 interviews per country. A random sample was used, with quotas for gender and age (16-24, 25-54, 55-74) to ensure a representative sample of participants. Table 1 summarizes the sampling information.

**Table 1:** Sampling information.

Population	Citizens aged from 16-74 years old who had used the Internet in the previous 3 months.
Geographical coverage	Austria, Belgium, Germany, Denmark, Estonia, Finland, France, Italy, Netherlands, Sweden, Slovenia, Slovakia, Spain, United Kingdom
Sample size	1000 interviews per country; 14,000 interviews in total
Quotas	Country; Gender (Female/Male); Age Group (16-24, 25-54, 55-74)
Sampling errors	+0.85% for overall data and +3.16% for country-specific data. In all cases, a maximum indeterminate probability (P=q=50), for a confidence level of 95.5% is applicable for each one of the reference populations.
Weighting	Proportional allocation for each country, to be able interpret the data at a country level; Weighting by population in each country to be able to interpret the overall data
Sampling	Individuals have been sampled in a completely random manner.

Table 2 summarizes the main sociodemographic characteristics. These results are broadly comparable to the characteristics of the Internet population in each country [30]. In order to interpret the overall data, country-specific differences have to be accounted for. The weighting factor was calculated by dividing the proportion of each country’s population to its total population by the proportion of individuals in each country’s sample to the total sample.

The main survey variable we were interested in was “Regarding health and Information and Communication Technologies, specifically the Internet, how often have you sent or received an email from your doctor, nurse or health care organization?” Participants were asked how often they had done this (every day/almost every day, at least once a week, at least every month, less than once a month, never, or I was not aware of it), and we split responses to this question into yes and no categories, with all the “never” and “I was not aware of it” responses comprising no, and the other groups merged to comprise yes. This allowed us to compare use and non-use of email with other key variables of interest. No definition of the term “email” was included in the survey. It is assumed that respondents understood the term in accordance with its use in common parlance.

**Table 2:** Sample sociodemographic characteristics (N=14,000).

		n (%)
**Gender**		
	Female^a^	7210 (51.50)
	Male^a^	6789 (48.50)
**Age**		
	16-24	2777 (19.84)
	25-54	8708 (62.20)
	55-74	2515 (18.96)
**Completed education**		
	Primary or lower secondary education (ISCED^b^ 0, 1, or 2)	2128 (15.20)
	Upper secondary education (ISCED^b^ 3 or 4)	6439 (45.99)
	Tertiary education (ISCED^b^ 5 or 6)	5433 (38.81)
**Employment status**		
	Employed or self-employed	8189 (58.49)
	Unemployed	1335 (9.54)
	Student (not in labor force)	2007 (14.34)
	Other (not in labor force)	2469 (17.64)

^a^n=13999; 1 do not know/did not answer.

^b^UNESCO International Standard.

### Statistical Analysis

Data analyses were completed using SPSS version 20.0. Chi-square tests were used to determine if there were any differences between use and non-use of email. An analysis of residuals was performed to determine the sources of significant findings. Under the null hypothesis that the 2 variables are independent, the adjusted residuals will have a standard normal distribution, that is, have a mean of 0 and standard deviation of 1. An adjusted residual that is more than 1.96 (2.0 is used by convention) indicates that the number of cases in that cell is significantly larger than would be expected if the null hypothesis were true, with a significant level of .05. An adjusted residual that is less than -2.0 indicates that the number of cases in that cell is significantly smaller than would be expected if the null hypothesis were true.

## Results

### Characteristics of Participants

Just over a quarter of participants (25.38%, 3553/14000) reported sending or receiving an email from their doctor, nurse or health care organization (Table 3). Participants largely reported good health: health status “good” or “very good” (74.29%, 10400/14000). Only 25.57% (3580/14000) reported not having a health problem; 58.11% (7849/13506) reported not experiencing any long-standing illness or health problem, and 65.87% (7849/13863) reported that they were not undergoing long-term medical treatment.

**Table 3:** Characteristics of participants.

Characteristics		n (%)
**Assessment of own health status**		
	Very bad	131 (0.94)
	Bad	891 (6.36)
	Neither good or bad	2578 (18.41)
	Good	7521 (53.72)
	Very good	2879 (20.56)
**Number of health problems reported**		
	None	3580 (25.57)
	1	3992 (28.51)
	2	3011 (21.51)
	More than 2	3417 (24.41)
**Current long-standing illness or health problem** ^a^		
	Yes	5657 (41.89)
	No	7849 (58.11)
**Undergoing long term medical treatment** ^b^		
	Yes	4732 (34.13)
	No	9131 (65.87)
**Number of visits to the doctor in the last 12 months** ^c^		
	None	1544 (11.03)
	1-2	4278 (30.56)
	3-4	2945 (21.04)
	5-6	2202 (15.73)
	More than 6	3030 (21.64)
**Sent or received an email from your doctor, nurse, or health care organization**		
	Yes	3553 (25.38)
	No	10447 (74.62)

^a^n=13,506; 494 do not know/did not answer.

^b^n=13,863; 137 do not know/did not answer.

^c^n=13,999; 1 do not know/did not answer.

### Country and Email Use

Of those reporting sending or receiving an email from their doctor, nurse, or health care organization, there was a statistically significant difference among countries: Denmark reported the highest level of emails sent/received at 50.70% (507/1000). The lowest level reported was by participants in France at 18.70% (187/1000). Respondents from Denmark, Estonia, Italy, and Sweden are more likely to use email within the context of health care than those in France, Belgium, Spain, Slovakia, Slovenia, and United Kingdom (Table 4).

**Table 4:** Sent/received an email from your doctor, nurse, or health care organization, by country.

Country^a,b^	Sent/received an email, n (%)	Adjusted residual
Austria	283 (28.30)	0.7
Belgium	222 (22.20)	-3.8
Germany	284 (28.40)	0.7
Denmark	507 (50.70)	17.1
Estonia	316 (31.60)	3.1
Finland	297 (29.70)	1.7
France	187 (18.70)	-6.4
Italy	363 (36.30)	6.5
Netherlands	253 (25.30)	-1.6
Spain	247 (24.70)	-2.0
Sweden	308 (30.80)	2.5
Slovakia	187 (18.70)	-6.4
Slovenia	198 (19.80)	-5.6
United Kingdom	186 (18.60)	-6.5

^a^Proportional allocation for each country (N=1000).

^b^χ^2^_13_=494.359; *P*=.000.

### Demographic Characteristics and Email Use

More men than women had used email (29.11%, 2099/7210) versus 21.42%, 1454/6789). Highest use was reported in the 16-24 age group (30.00%, 833/2777) and in those educated to tertiary level (27.00%, 1467/5433). Lowest use was reported in the 55-74 age group (20.16%, 507/2515) and in those educated to primary or lower secondary level (22.98%, 489/2128). Students reported the highest level of use (28.95%, 581/2077), with the lowest levels reported by those in the “Other” group, that is, people outside of the labor force (20.66%, 510/2468) (Table 5).

**Table 5:** Sent/received an email from your doctor, nurse, or health care organization, by demographic characteristic

Characteristics		Sent/received an email	
		Yes, n (%) adjusted residual	No, n (%) adjusted residual
**Gender** ^a^			
	Female	1454 (21.42) -10.5	5335 (78.58) 10.5
	Male	2099 (29.11) 10.5	5111 (70.89) -10.5
**Age** ^b^			
	16-24	833 (30.00) 6.2	1944 (70.00) -6.2
	25-54	2213 (25.41) 0.1	6495 (74.59) -0.1
	55-74	507 (20.16) -6.6	2008 (79.84) 6.6
**Completed education** ^c^			
	Primary or lower secondary education (ISCED^d^ 0, 1, or 2)	489 (22.98) -2.8	1639 (77.02) 2.8
	Upper secondary education (ISCED^d^ 3 or 4)	1598 (24.81) -1.4	4842 (75.19) 1.4
	Tertiary education (ISCED^d^ 5 or 6)	1467 (27.00) 3.5	3966 (73.00) -3.5
**Employment status** ^e^			
	Employed or self employed	2184 (26.67) 4.2	6005 (73.33) -4.2
	Unemployed	278 (20.82) -4.0	1057 (79.18) 4.0
	Student (not in labor force)	581 (28.95) 4.0	1426 (71.05) -4.0
	Other (not in labor force)	510 (20.66) -5.9	1958 (79.34) 5.9

^a^χ^2^_1_=109.332; *P*=.000.

^b^χ^2^_2_=67.455; *P*=.000.

^c^χ^2^_2_=15.109; *P*=.001.

^d^UNESCO International Standard.

^e^χ^2^_3_=64.299; *P*=.000.

### Health Status, Health Resource Utilization, and Email Use

The highest level of email use is reported in those who state that their general health is very bad (40.46%, 53/131). Respondents with more than 2 health problems also report the highest level of email use (33.63%, 1149/3417), indicating that the poorer a person’s health, the more likely they are to have used email in this way. As self-reported health state worsens, the proportion of people reporting having sent or received an email increases (Table 6).

As seen in Table 6, the relationship between email use for health care and number of visits to the doctor was varied. Over 60% of respondents who reported visiting the doctor 5-6 times and more than 6 times in the last 12 months had also communicated with their health care provider by email. Those who reported having more than 6 visits in the last 12 months reported the highest use of email (30.33%, 919/3030). Those who reported not visiting the doctor at all in the preceding 12 months reported the lowest level of email use (15.54%, 240/1544). As visit number increases, so does the proportion of people reporting having sent or received an email and vice versa.

**Table 6:** Sent/received an email from your doctor, nurse, or health care organization, by health status and health care utilization.

		Sent/received an email	
		Yes, n (%) adjusted residual	No, n (%) adjusted residual
**Assessment of own health status** ^a^			
	Very bad	53 (40.46) 4.0	78 (59.54) -4.0
	Bad	292 (32.77) 5.2	599 (67.23) -5.2
	Neither good or bad	680 (26.38) 1.3	1898 (73.62) -1.3
	Good	1863 (24.77) -1.8	5658 (75.23) 1.8
	Very good	665 (23.10) -3.2	2214 (76.90) 3.2
**Number of health problems reported** ^b^			
	None	761 (21.25) -6.6	2820 (78.75) 6.6
	1	890 (22.29) -5.3	3103 (77.71) 5.3
	2	754 (25.04) -0.5	2257 (74.96) 0.5
	More than 2	1149 (33.63) 12.7	2268 (66.37) -12.7
**Current long-standing illness or health problem** ^c^			
	Yes	1665 (29.82) 9.0	3991 (70.56) -9.0
	No	1771 (22.56) -9.0	6078 (77.44) 9.0
**Undergoing long term medical treatment** ^d^			
	Yes	1411 (29.82) 8.9	3321 (70.18) -8.9
	No	2090 (22.89) -8.9	7040 (77.11) 8.9
**Number of visits to the doctor in the last 12 months** ^e^			
	None	240 (15.54) -9.4	1304 (84.46) 9.4
	1-2	1003 (23.44) -3.5	3276 (76.56) 3.5
	3-4	736 (24.99) -0.6	2209 (75.01) 0.6
	5-6	656 (29.78) 5.2	1547 (70.22) -5.2
	More than 6	919 (30.33) 7.1	2111 (69.67) -7.1

^a^χ^2^_4_=52.178; *P*=.000.

^b^χ^2^_3_=175.235; *P*=.000.

^c^χ^2^_1_=81.893; *P*=.000.

^d^χ^2^_1_=79.214; *P*=.000.

^e^χ^2^_4_=149.294; *P*=.000.

## Discussion

### Summary

This study provides the first Europe-wide exploration of email use by patients for health care communication purposes. The most prolific users of email were men, those aged 16-24, and students. Higher numbers of people in poor health used email relative to those reporting good health and no ongoing conditions.

### Country and Email Use

The high level of email communication reported in Denmark is consistent with their health policy. It is compulsory for all doctors in Danish primary care services to offer their patients email contact and online services, and structures are in place to provide reimbursement for this use. The Danish public national health portal, Sundhed.dk, has been established for more than a decade and successfully integrates a variety of features with the explicit aim of both facilitating the smooth delivery of national health care priorities and maintaining Denmark’s reputation as a world leader in the digital health arena [12]. The high levels of use in Denmark are not typical of European implementation. The level of use reported in this study for the United Kingdom (18.6) is similar to prevalence estimates in other UK-based surveys that currently estimate use in general practice settings at between 20-23% [31]. The use of email by country is hugely varied and indicative of hugely disparate eHealth communication priorities and strategies among the European countries surveyed.

### Demographic Characteristics and Email Use

Highest use of email for health care was reported among the youngest and most educated groups, and lowest use was reported among the oldest, least educated, and unemployed respondents. These findings corroborate the general perception that certain groups are deemed to be at an immediate digital disadvantage and may be disenfranchized as a result. The higher use of email by men in the sample is a case in point and is a particularly interesting finding as it contests the comprehensive perception that women are more likely to use the Internet for health care purposes. As eHealth research becomes increasingly nuanced, evidence of gender differences in the context of particular health-related online behaviors becomes apparent. The perceived importance of online health information as a credible resource is indeed particularly strong among young women [1] and yet, in this study, significantly more men used the opportunity to actively communicate with a health care professional by email.

### Health Status, Heath Resource Utilization, and Email

Concerns that opportunities for email communication encourage inappropriate use by the “worried well” were unfounded among this sample. Health status was consistently negatively associated with email use, with over 40% of those who rated their health as being very bad having used email to communicate with a health professional, compared to just over 23% of those who rated their health as being very good. This may be due to increased need for contact with health care services among those people with poor health and possibly reflects email being used as an alternative to other forms of contact. There is also the possibility that it reflects a desire by those with multimorbidity to have repeated and frequent contact with their health care professional. People living with a chronic condition who have access to the Internet are significantly more likely than other online adults to gather and act upon health-related behavior [2].

### Strengths and Limitations

This research provides key insights into the use of email for health care communication in Europe, particularly in the context of establishing impact on health care resource use. The specific value of email communication to specific groups of people is clear, despite overall use of email for health care communication being relatively low. It should be noted that low use of email does not necessarily indicate low interest. Research exploring expectations of the future use of digital health services consistently highlights substantial interest among European citizens in using email for health care but also stresses that this interest comes with reservations, largely around the ability of local technical infrastructure to cope, patient confidentiality, and a negative perception of clinicians’ willingness to use alternative forms of communication [32]. Indeed, in countries where the implementation of a coherent digital health strategy has been particularly slow (eg, Poland), interest remains high but expectation has fallen sharply [6].

These data feature only responses from digitally literate individuals. So although we are able to describe email use only among this population of Internet users, we know that across the European countries included, some 73% of people are online [30] and so the survey covers a large proportion of the population. Coupled with the quota sampling approach, the sample is as representative as can be expected for an online survey. The age range of participants was 16-74 years, thus people in the very oldest age groups were excluded. As these are also likely to be lowest users of the Internet by age group, it is possible that the impact of the digital divide is underestimated in this study.

There is likely to be some confounding of the results because we were looking at so many different factors, and it is important to view these results within the scope of what is possible to ascertain through secondary analysis of questionnaire data. Potential confounds may not be related to demographics or may be related to variables not addressed by the questionnaire at all. Without further research, it is unwise to extrapolate. Sample size means that it is not surprising that there was a significant difference between groups; they may reflect differences between groups, but not necessarily meaningful differences. The variable of interest was not designed as a yes/no question in the survey, and so the results must be viewed in light of this. The “no” includes both people actively deciding not to use it, and those who were not aware of it. The “yes” group includes people having used it at varying frequencies. The two groups in each category may be very different. The variable itself includes communication with a doctor, nurse, or health care organization. Respondents’ understanding of the term “email” may have differed. In addition, this contact may be disparate, with emails to a doctor or nurse being different in nature than those to an organization. However, it is presumed that intention to use the technology for communication relating to health care would be the same, as would associated processes.

### Conclusions

We can conclude that the use of email for communication in the context of health care is of particular value to specific groups of patients despite relatively low use. Additionally, low use does not necessarily indicate a lack of interest or willingness to engage with health care in this way. Increased use of email is not associated with increased visits to a physician among the “worried well” but is associated with increased overall virtual and physical engagement among those with chronic and multimorbid conditions. Qualitative studies need to be conducted in order to develop our understanding of this phenomenon. Recognizing and understanding the nuances of email communication is crucial in ensuring that any use of email in health care is equitable. It is important to develop guidance around best practice in the use of email, and there are clear opportunities for communities and countries to learn from each other’s success. Implemented carefully, email communication could become an important tool for health care professionals, which may allow aspects of consultation to move beyond traditional settings.
